# Prognostic prediction of preoperative nutritional status in low-grade appendiceal mucinous neoplasms

**DOI:** 10.1038/s41598-022-14765-y

**Published:** 2022-06-23

**Authors:** Bing Wang, Ruiqing Ma, Guanjun Shi, Zhenpeng Yang, Huazhen Tang, Shuai Lu, Yuying Wang, Jinxiu Qu, Benqiang Rao, Hongbin Xu

**Affiliations:** 1 Department of Gastrointestinal Surgery, Beijing Shijitan Hospital, Capital Medical University, Beijing, 100038 China; 2 Department of Myxoma, Aerospace Center Hospital, Beijing, 100049 China

**Keywords:** Cancer, Diseases, Oncology, Risk factors

## Abstract

To describe the preoperative nutritional status of Low-grade Appendiceal Mucinous Neoplasms (LAMNs) and identify prognostic factors for survival. Medical records from 165 patients with LAMNs who attended the Aerospace Center Hospital, Beijing, China between January 2017, and December 2018 were retrospectively reviewed. Survival analysis was performed with the Kaplan–Meier method, the log-rank test, and a Cox proportional hazards model. Among 165 patients, 59 (36%) were male and 106 (64%) were female. Patient’s median age was 58 years (range 20 to 78 years). Univariate analysis indicated that gender, weight loss, prior surgical score (PSS), red blood cell, albumin, peritoneal cancer index (PCI), completeness of cytoreduction (CCR), and hyperthermic intraperitoneal chemotherapy (HIPEC) were related to prognosis. Multivariate analysis showed that PSS, CCR and HIPEC were independent predictors of prognosis. The preoperative nutritional status of patients plays an important role in predicting prognosis. Patients can benefit from a complete cytoreductive surgery (CCRS) and HIPEC in an experienced institution for the first medical treatment.

## Introduction

Pseudomyxoma peritonei (PMP) is a rare malignant neoplasm, which is characterized by mucinous masses and or ascites in the abdominal cavity. It is usually a primary mucinous neoplasm originating from the appendix^1^. In 1842, Karl F. Rokitansky first reported mucocele of appendix^2^. The combination of cytoreductive surgery (CRS) and hyperthermic intraperitoneal chemotherapy (HIPEC) has been regarded as standard of care for PMP^3^. The clinical manifestations of most patients are anorexia and weight loss. There are few and unsystematic reports on whether the preoperative nutritional status can predict the prognosis. Based on this, our study retrospectively analyzed the clinical data to find the prognostic factors, so as to solve the problems concerned by patients and provide basis for clinical treatment.

## Patients and methods

### Ethical approval of the study

The Ethics Committee of the Aerospace Center Hospital, Beijing, China (No. 20161109-ST-07) approved this study. All methods were performed in accordance with the relevant guidelines and regulations. Informed consent was obtained from all participants and/or their legal guardians in this study. The research has been performed in accordance with the Declaration of Helsinki.

### Patients

Medical records from a database of patients with PMP who attended the Aerospace Center Hospital, Beijing, China between January 2017 and December 2018 were retrospectively reviewed. Inclusion criteria were: (1) diagnosis of Low-grade Appendiceal Mucinous Neoplasms (LAMNs) on histology and histopathologic subtype confirmed by two experienced pathologists; and (2) treatment with CRS and HIPEC. Exclusion criteria were: (1) PMP derived from other organs or other pathological types; (2) loss to follow-up; (3) incomplete medical records; or (4) history of other severe organic disease. A total of 165 patients were included in the final analysis.

### Surgery treatment

Patients underwent CRS to remove visible tumors if any^4^. HIPEC was conducted in patients for 60 min using a closed-abdomen technique with cisplatin 60 ~ 80 mg and an extracorporeal device that maintained intraabdominal temperature between 40 and 42 °C.

### Study parameters

The analysis included the following clinicopathological parameters: gender, age, intestinal obstruction, weight loss, prior surgical score (PSS), red blood cell (RBC), hemoglobin (Hb), albumin (ALB), hospitalization, right hemicolectomy, intraoperative peritoneal cancer index (PCI), completeness of cytoreduction (CCR), operation time, blood loss volume, intraoperative RBC transfusion volume, intraoperative plasma transfusion volume, intraoperative HIPEC, and follow-up time. We obtained cutoff values based on objective data for gender, intestinal obstruction, weight loss, PSS, RBC, Hb, ALB, right hemicolectomy and HIPEC, among which the cutoff values for Hb and ALB were based on the lower limit of the normal reference range. The cutoff values of age, PCI, and CCR were obtained according to the previous grouping of our research center^5–8^. Due to the wide range of hospitalization days, we selected one month as the cutoff value. We used 1000 ml as the cutoff value of blood loss according to the indication for blood transfusion. We used the median to obtain cutoff values for operation time, intraoperative RBC and plasma transfusion volume.

Among these characteristics, PSS was scored on a scale from 0 to 3, where PSS-0 was no surgery or biopsy had been performed for cancer, PSS-1 was surgery had been performed in one abdominal region, PSS-2 was surgery had been performed in two to five regions, and PSS-3 was surgery had been performed in > 5 regions^9^. The PCI was determined intraoperatively where neoplasm volume and extent of peritoneal dissemination in each of 13 abdominopelvic regions (nine anatomical regions in the abdomen and four segments in the small bowel) were scored on a scale from 0 to 3 and summed^4^. Residual disease following CRS was scored according to the CCR on a scale from 0 to 3, where CCR-0 was no macroscopic residual cancer remained, CCR-1 was residual neoplasm nodules < 2.5 mm remained, CCR-2 was nodules between 2.5 mm and 2.5 cm remained, and CCR-3 was persistent neoplasm nodules > 2.5 cm remained^10^. Overall survival (OS) was calculated from the date of first CRS and HIPEC to the time of death or the last follow-up.

### Follow-up

The patients were reexamined every 6 months, including abdominopelvic enhanced CT and tumor markers. The follow-up method was telephone and or reexamine. The follow-up time was from the date of first surgical diagnosis to June 2020, and OS was counted. All patients were followed up.

### Statistical analysis

Statistical analyses were conducted using SPSS version 20 (SPSS, Chicago, Illinois, USA). Continuous data are presented as medians and range. Categorical data are presented as number and percentages. Univariate survival analysis was performed with the Kaplan–Meier method and the log-rank test. Statistically significant variables were included in a multivariate analysis, which used a Cox proportional hazards model to identify independent prognostic factors for survival. All live patients were censored. P < 0.05 was considered statistically significant.

### Ethics approval and consent to participate

This is a retrospective study, and all the patients signed the consent for treating before the treatment. Additionally, the participants have given the consent for their images. The study was approved by the Ethic committee of Aerospace Central Hospital, Beijing, China (no.20161109-ST-07).

### Consent for publication

Written informed consent was given by the participants, and all the participants have given the consent for their images.

## Results

### Clinical data characteristics

From January 2017 to December 2018, 235 patients had appendix derived mucinous neoplasms underwent CRS and HIPEC. A total of 165 patients with LAMNs had CRS and HIPEC. All patients were followed up and the clinical data were complete.

Demographic and clinical characteristics of the 165 included patients are presented in Table 1. Among 165 patients, 59 (36%) were male and 106 (64%) were female. Patient’s median age was 58 years (range 20–78 years). Twenty-seven (16%) patients had preoperative intestinal obstruction and 64 (39%) patients had weight loss before operation. The patients with PSS scores of 0, 1, 2 and 3 were 40 (24%), 40 (24%), 45 (28%) and 40 (24%) respectively. Preoperative erythrocyte abnormalities, anemia, albumin decreased in 77 (47%), 66 (40%) and 60 (36%) patients, respectively.

**Table 1:** Patients’ clinical and demographic data (n = 165).

Characteristics	No. of patients
**Gender**	
Male	59 (36%)
Female	106 (64%)
**Age**	
Median (range)	58 (20–78)
< 50	38 (23%)
≥ 50	127 (77%)
**Intestinal obstruction**	
Yes	27 (16%)
No	138 (84%)
**Weight loss**	
Yes	64 (39%)
No	101 (61%)
**PSS**	
0	40 (24%)
1	40 (24%)
2	45 (28%)
3	40 (24%)
**RBC**	
Normal	88 (53%)
Abnormal	77 (47%)
**Hb (g/L)**	
< 110	66 (40%)
≥ 110	99 (60%)
**ALB (g/L)**	
< 35	60 (36%)
≥ 35	105 (64%)
**Hospitalization (days)**	
Median (range)	26 (16–47)
< 30	127 (77%)
≥ 30	38 (23%)
**Right hemicolectomy**	
Yes	120 (73%)
No	45 (27%)
**PCI**	
Median (range)	32 (0–39)
< 20	28 (17%)
≥ 20	137 (83%)
**CCR**	
0–1	50 (30%)
2–3	115 (70%)
**Operation time (min)**	
Median (range)	420 (210–860)
< 420	23 (14%)
≥ 420	142 (86%)
**Blood loss volume (ml)**	
Median (range)	1500 (100–7000)
< 1000	29 (18%)
≥ 1000	136 (82%)
**Intraoperative RBC transfusion volume (U)**	
Median (range)	4 (0–22)
< 4	62 (38%)
≥ 4	103 (62%)
**Intraoperative plasma transfusion volume (ml)**	
Median (range)	400 (0–2200)
< 400	51 (31%)
≥ 400	114 (69%)
**HIPEC**	
Yes	138 (84%)
No	27 (16%)

*PSS* prior surgical score, *RBC* red blood cell, *Hb* hemoglobin, *ALB* Albumin, *PCI* peritoneal cancer index, *CCR* completeness of cytoreduction, *HIPEC* hyperthermic intraperitoneal chemotherapy.

Intraoperative PCI was < 20 and ≥ 20 in 28 (17%) and 137 (83%) patients, respectively. CCR score was 0/1 and 2/3 in 50 (30%) and 115 (70%) patients, respectively. Operation time was < 420 min and ≥ 420 min in 23 (14%) and 142 (86%) patients, respectively. Blood loss volume was < 1000 ml and ≥ 1000 ml in 29 (18%) and 136 (82%) patients, respectively. Intraoperative RBC transfusion volume was < 4U and ≥ 4U in 62 (38%) and 103 (62%) patients, respectively. Intraoperative plasma transfusion volume was < 400 ml and ≥ 400 ml in 51 (31%) and 114 (69%) patients, respectively. The patients with right hemicolectomy, hospital stay ≥ 30 days and HIPEC were 120 (73%), 38 (23%) and 138 (84%) respectively. All these factors were analyzed by univariate analysis, and the statistically significant results were analyzed by multivariate analysis.

### Prognostic factors analysis

**Figure 1:**
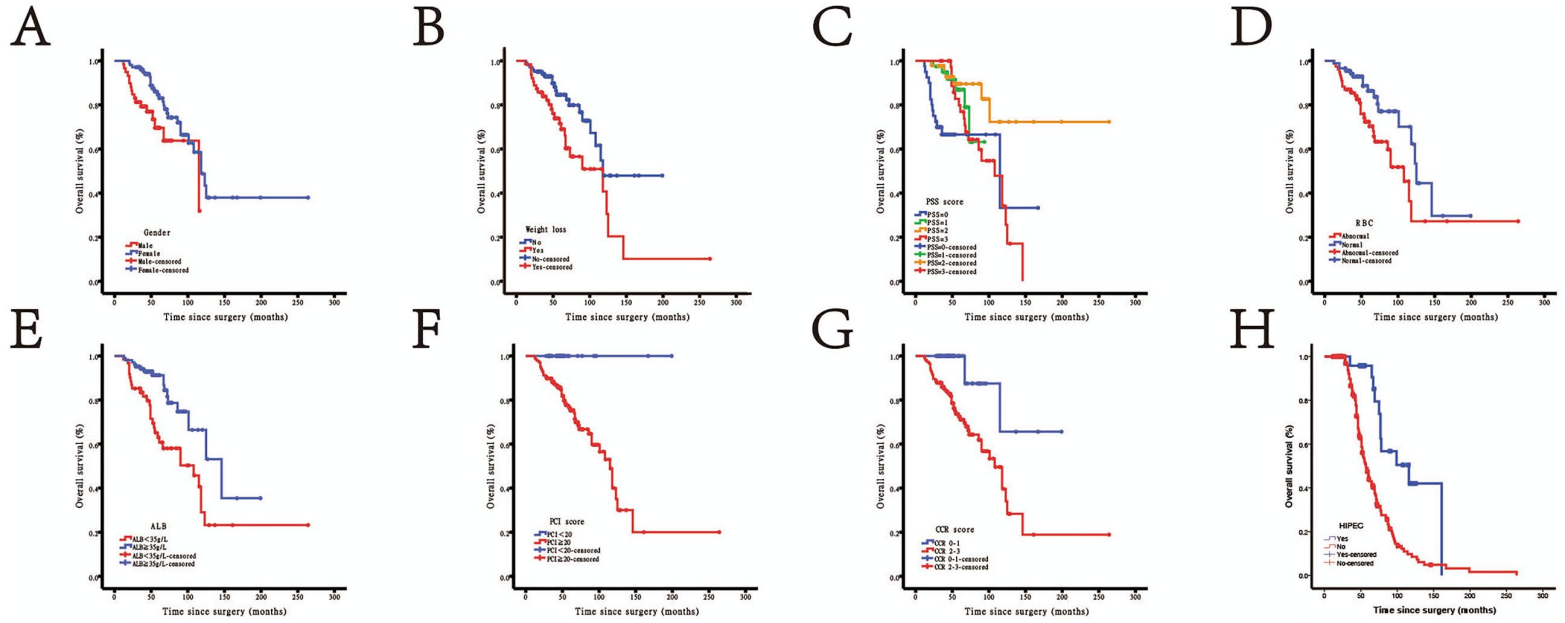
Kaplan–Meier survival curves for gender (**A**), weight loss (**B**), PSS (**C**), RBC (**D**), ALB (**E**), PCI (**F**), CCR (**G**), and HIPEC (**H**) (n = 165).

Prognostic factors for OS on univariate analysis are presented in Table 2. There are eight factors related to prognosis among the seventeen research factors, namely gender (male vs. female, P = 0.015), weight loss (yes vs. no, P = 0.007), PSS (0, 1, 2, 3, P = 0.001), RBC (abnormal vs. normal, P = 0.016), ALB (< 35 vs. ≥ 35, P = 0.001), PCI (≥ 20 vs. < 20, P = 0.004), CCR (2/3 vs. 0/1, P = 0.001), and HIPEC (no vs. yes, P < 0.001). All factors were prognostic for OS by the log rank test in patients with LAMNs (Fig. 1). Multivariate analysis showed that PSS, CCR and HIPEC were independent predictors of prognosis (Table 3).

**Table 2:** Univariate analysis of OS after CRS (n = 165).

Variables	HR (95% CI)	P value
Gender (male vs. female)	0.455 (0.240–0.862)	0.015
Age (< 50 years vs. ≥ 50 years)	1.021 (0.990–1.053)	0.730
Intestinal obstruction (yes vs. no)	1.589 (0.841–3.003)	0.111
Weight loss (yes vs. no)	2.170 (1.210–3.889)	0.007
PSS (0 vs. 1 vs. 2 vs. 3)	0.472 (0.265–0.702)	0.001
RBC (abnormal vs. normal)	0.632 (0.388–1.029)	0.016
Hb (< 110 g/L vs. ≥ 110 g/L)	0.990 (0.975–1.005)	0.497
ALB (< 35 g/L vs. ≥ 35 g/L)	0.536 (0.318–0.829)	0.001
Hospitalization (≥ 30 days vs. < 30 days)	1.011 (0.961–1.064)	0.645
Right hemicolectomy (yes vs. no)	1.907 (0.950–3.826)	0.324
PCI (≥ 20 vs. < 20)	2.071 (1.160–3.925)	0.004
CCR (2–3 vs. 0–1)	2.651 (1.535–4.576)	0.001
Operation time (< 420 min vs. ≥ 420 min)	1.000 (0.997–1.002)	0.406
Blood loss volume (≥ 1000 ml vs. < 1000 ml)	1.021 (1.001–1.227)	0.135
Intraoperative RBC transfusion volume (< 4U vs. ≥ 4U)	1.000 (0.943–1.006)	0.359
Intraoperative plasma transfusion volume (< 400 ml vs. ≥ 400 ml)	1.000 (0.967–1.180)	0.607
HIPEC (no vs. yes)	2.752 (1.609–4.805)	< 0.001

*OS* overall survival, *CRS* cytoreductive surgery, *HR* hazard ratio, *CI* confidence interval, *PSS* prior surgical score, *RBC* red blood cell, *Hb* hemoglobin, *ALB* Albumin, *PCI* peritoneal cancer index, *CCR* completeness of cytoreduction, *HIPEC* hyperthermic intraperitoneal chemotherapy.

**Table 3:** Multivariate analysis for statistically significant results (n = 165).

**Variables**	B	SE	Wald	df	Sig	Exp(B)	95.0% CI for Exp(B)	
							Bottom	Upper
PSS	− 0.422	0.148	8.083	1	0.004	0.656	0.490	0.877
HIPEC	0.952	0.277	11.782	1	0.001	2.591	1.504	4.463
CCR	1.080	0.278	15.102	1	0.000	2.945	1.708	5.078

*PSS* prior surgical score, *HIPEC* hyperthermic intraperitoneal chemotherapy, *CCR* completeness of cytoreduction.

## Discussion

In 2016, the Peritoneal Surface Oncology Group International (PSOGI) classified appendiceal mucinous neoplasms into four categories: low-grade appendiceal mucinous neoplasms (LAMNs), high-grade appendiceal mucinous neoplasms (HAMNs), Mucinous adenocarcinoma (MAC) and mucinous adenocarcinoma with signet ring cells (MAC-S)^11^. Their definition on appendiceal mucinous epithelia are regarded as a milestone^12^. All patients included in this study were pathologically confirmed as low-grade mucinous neoplasms of appendiceal origin. In order to avoid the possible outcome bias caused by pathological types, this study only analyzed the characteristics of patients with the most common LAMNs.

This study described the perioperative risk factors affecting the prognosis of LAMNs and identified the independent predictors of OS. From January 2017 to December 2018, 235 patients with appendiceal mucinous neoplasms underwent surgery in Aerospace Center Hospital, Beijing, China. PMP is a clinical syndrome characterized by "jelly like" neoplasms formed by mucinous neoplasms implanted on the surface of surrounding organs. PMP mainly originates from the appendix, which is consistent with other studies^13^. Our study was designed according to different pathological types, of which 165 patients were included in our analysis.

Our center is one of the major centers for the treatment of pseudomyxoma peritonei in China. Due to China's large population base, the number of people suffering from this disease is relatively large. But many patients do not have their first operation in our center when they are diagnosed. The most patients had a large neoplasm load and were located in the middle or late stage of the disease when sought medical advice. The median PCI score was 32 (range, 0 to 39) which was the reason for the low radical rate, and the complete CRS was maintained at about 30%. Although univariate analysis showed that PCI could predict prognosis, it was not an independent predictor of OS after CRS and HIPEC. On the contrary, CCR can be used as an independent predictor of OS after CRS and HIPEC, suggesting that patients will benefit from reaching CCR 0–1. However, whether the right hemicolectomy was removed or not, operation time, blood loss volume, intraoperative RBC infusion volume or plasma volume and length of hospital stay could not predict postoperative survival. Despite this, we recommend that a definitive follow-up schedule be formulated after CRS and HIPEC to detect recurrence and plan subsequent treatment.

We scored the prior surgical outcomes with PSS for each patient; PSS-0 was the rating for no surgery or biopsy, PSS-1 for surgery in one abdominal region only, PSS-2 for surgery in two to five regions, and PSS-3 for surgery in more than five regions^5^. A recent report showed that the univariate analysis of poor prognosis in patients with peritoneal pseudomyxoma after neoplasm reduction included women (P = 0.0127), intestinal obstruction before treatment (P = 0.00791) and PSS (P = 0.0054). There was also a significant difference in PSS in multivariate analysis (P = 0.0041)^14^. Our results suggested that male postoperative survival was worse, but it was not an independent factor for the prognosis. We have not confirmed that intestinal obstruction before surgery was related to postoperative survival, which may be relevant to most of the selected cases were incomplete intestinal obstruction. Our results also showed that PSS can be used as an independent predictor of OS after CRS and HIPEC, suggesting that the more times of operations, the poorer prognosis. On the contrary, patients can benefit from the first diagnosis and treatment to an experienced institution to complete the maximum degree of neoplasm reduction surgery.

We creatively studied the general indicators of preoperative nutritional status, including weight loss, erythrocyte count, anemia and ALB, and analyzed the relationship between them and postoperative survival. Univariate analysis showed that weight loss, RBC count decrease and ALB decrease were related to poor postoperative survival, but they were not independent predictors, indicating that the general state of patients before operation was closely related to the prognosis. Anemia had nothing to do with postoperative survival, which may be related to our sufficient blood transfusion, and it also showed that RBC can better predict postoperative survival than hemoglobin. A study of modified Glasgow Prognostic Score (mGPS) predicting survival after complete CRS and HIPEC in patients with appendiceal peritoneal pseudomyxoma. The mGPS for each patient was calculated using the C-reactive protein (CRP) and albumin levels^15^. The mGPS system consists of 3 scores (0, 1, 2); patients without elevation of CRP (≤ 10 mg/L) are classified as mGPS-0 regardless of their albumin level. Patients with elevated CRP levels > 10 mg/L and normal albumin are classified as mGPS-1, and patients with elevated CRP and hypoalbuminaemia (< 35 g/L), are classified as mGPS-2^16^. The results showed that the long-term survival rate decreased along with the increase of mGPS^17^. This also indirectly reflects the value of albumin in predicting postoperative survival of patients with PMP.

The combination of CRS and HIPEC is recognized as the gold standard treatment for appendix derived PMP^18^. One study showed that higher PCI predicted lower progression free survival in patients with appendiceal PMP, and the impact of PCI remained an important prognostic variable in patients with low-grade appendiceal pseudomyxoma and appendiceal adenocarcinoma^9^. The study also demonstrated that a higher CCR score (CCR-2 or CCR-3) was associated with significantly worse 5-year survival (24%), compared with 85% in patients with CCR-0 and 80% in patients with CCR-1. In our study, through univariate analysis, CCR (2/3 vs. 0/1) and PCI (≥ 20 vs. < 20) were the prognostic factors of OS in LAMNs patients. We have reason to assume that early detection, early treatment and maximum cell reduction will achieve better long-term survival. Another multicenter cohort study analyzed 1924 patients with PMP due to an appendiceal mucinous neoplasm. Their results showed that compared with the CRS-alone group, the CRS-HIPEC group had better 5-year overall survival in all subsets, including CC-2/3 (16.1% [95% CI 10.4–24.8%] vs 28.4% [95% CI 19.6–41.1%]; P = 0.007)^19^. Our study also showed that with or without HIPEC was an independent prognostic factor of LAMNs, suggesting that patients will benefit from HIPEC on the premise that they can tolerate HIPEC.

## Conclusions

In conclusions, this study systematically analyzes of the prognostic factors of perioperative LAMNs patients, especially the analysis of some preoperative indicators that reflect the general condition of patients. It is innovative compared with the previous studies. PSS and CCR can be used as independent predictors of the prognosis of LAMNs patients, which provide a theoretical basis for judging the prognosis of patients before clinical operation, and also draw the importance of complete CRS in an experienced institution for the first medical treatment. However, other pathological types and prospective studies need to further verify the situation for patients.

## Data Availability

The datasets used and/or analyzed during the current study are available from the corresponding author on reasonable request.

## Abbreviations

PMP: Pseudomyxoma peritonei
CRS: Cytoreductive surgery
HIPEC: Hyperthermic intraperitoneal chemotherapy
PSS: Prior surgical score
RBC: Red blood cell
Hb: Hemoglobin
ALB: Albumin
PCI: Peritoneal cancer index
CCR: Completeness of cytoreduction
OS: Overall survival
HR: Hazard ratio
CI: Confidence interval
LAMNs: Low-grade appendiceal mucinous neoplasms
HAMNs: High-grade appendiceal mucinous neoplasms
MAC: Mucinous adenocarcinoma
MAC-S: Mucinous adenocarcinoma with signet ring cells
mGPS: Modified Glasgow Prognostic Score
CRP: C-reactive protein
